# Supplementary material for: Predicting Prognosis and Immunotherapy Response in Multiple Cancers Based on the Association of PANoptosis-Related Genes with Tumor Heterogeneity
Source: Genes (Basel). 2023 Oct 25;14(11):1994. doi: 10.3390/genes14111994 (PMC10671595; doi:10.3390/genes14111994)
Supplement: Supplementary file 1 [file genes-14-01994-s001.zip › supplemental data.pdf]

Supplemental Data

Supplementary Figure S1

A LGG+GBM

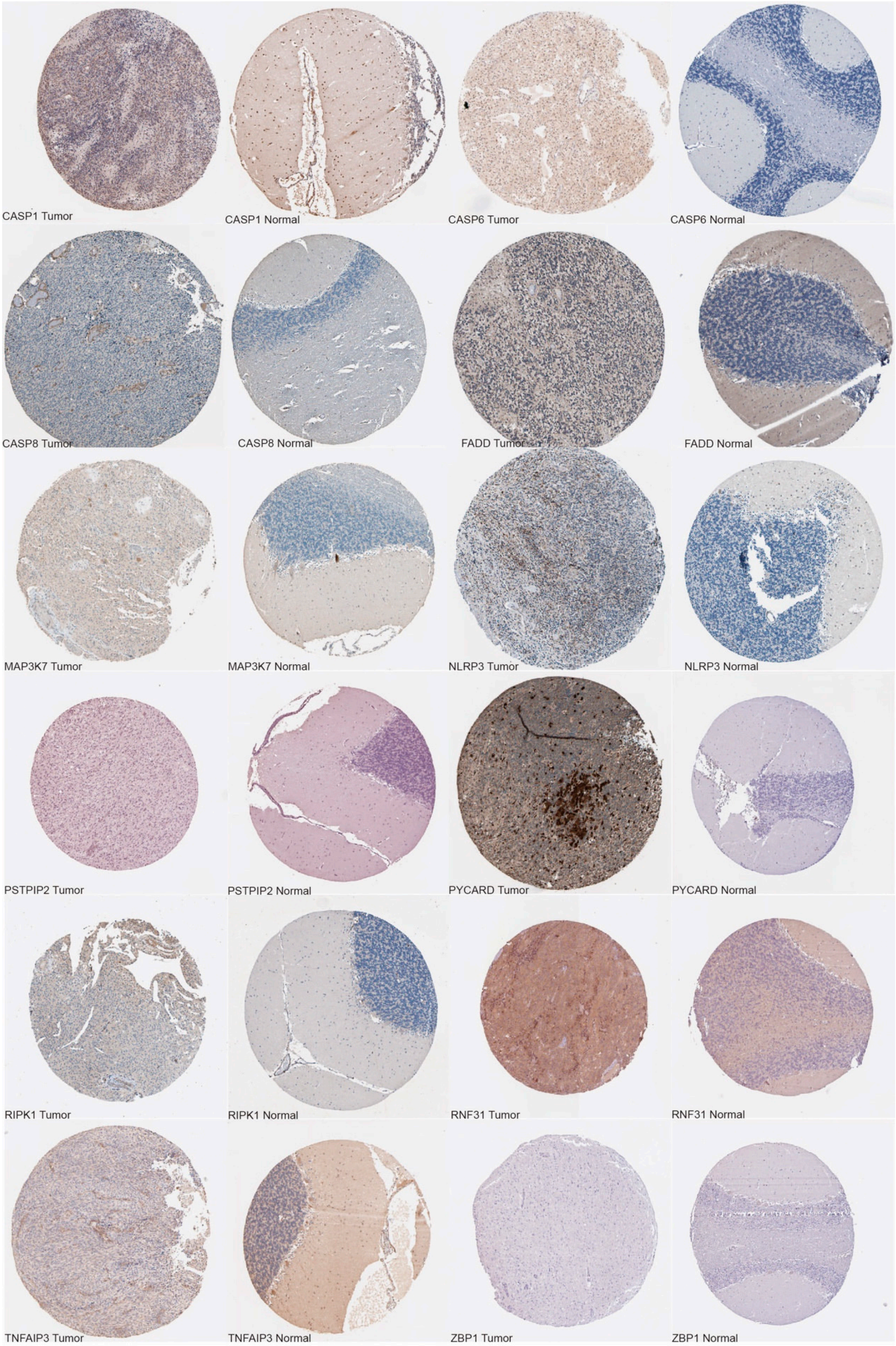

B KIRC

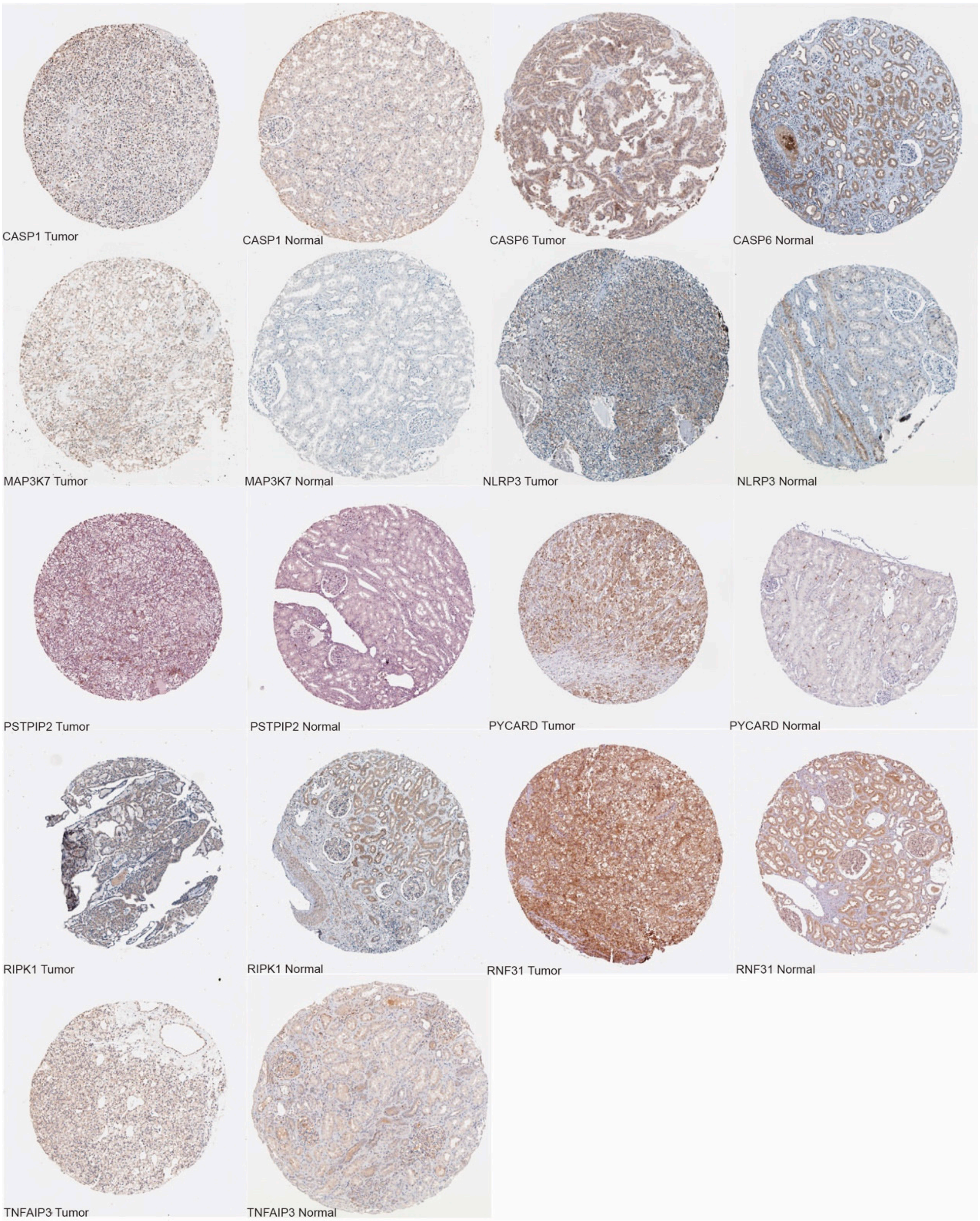
CASP1 Tumor
CASP1 Normal
CASP6 Tumor
CASP6 Normal
MAP3K7 Tumor
MAP3K7 Normal
NLRP3 Tumor
NLRP3 Normal
PSTPIP2 Tumor
PSTPIP2 Normal
PYCARD Tumor
PYCARD Normal
RIPK1 Tumor
RIPK1 Normal
RNF31 Tumor
RNF31 Normal
TNFAIP3 Tumor
TNFAIP3 Normal

C PAAD

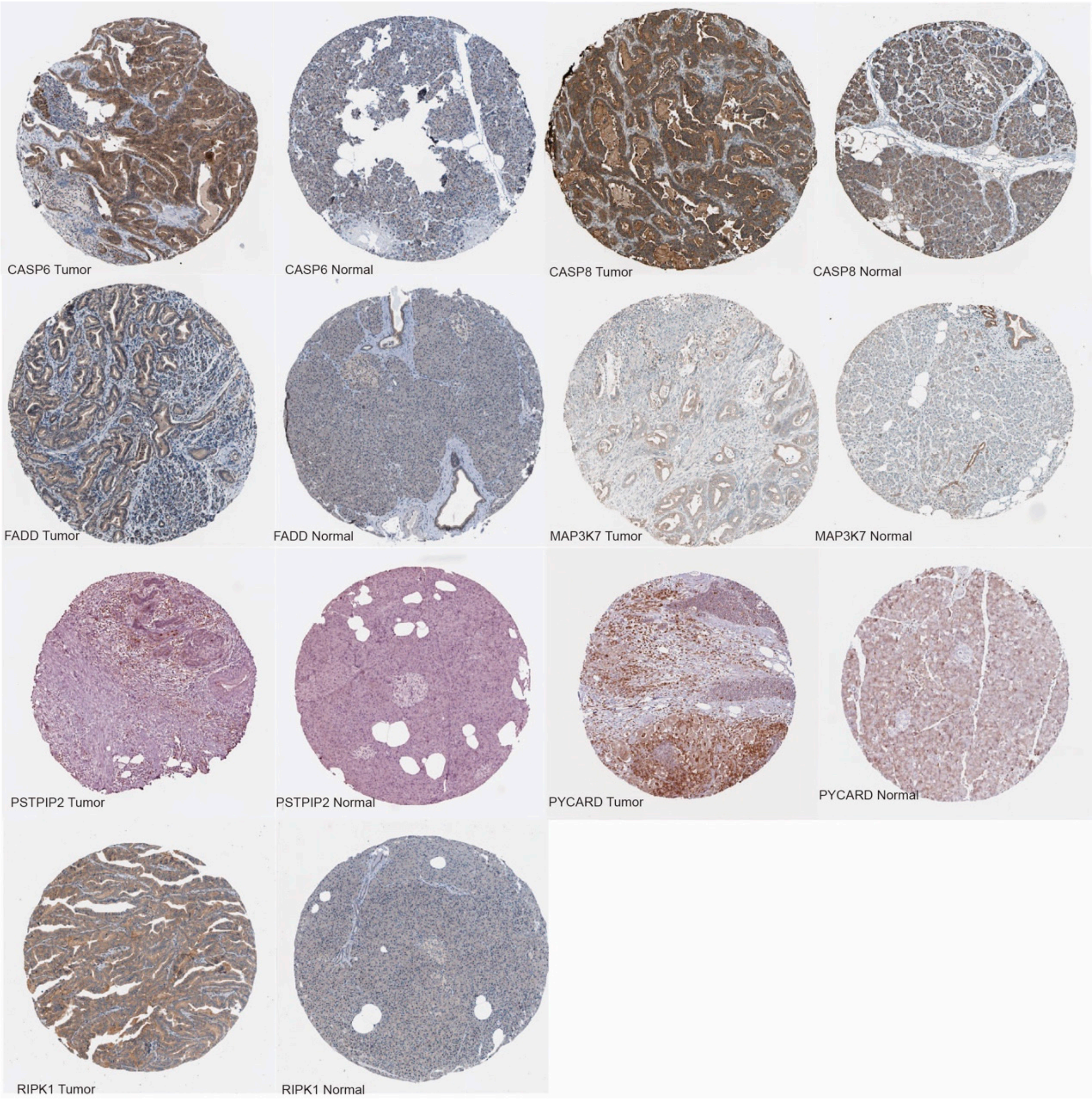
CASP6 Tumor
CASP6 Normal
CASP8 Tumor
CASP8 Normal
FADD Tumor
FADD Normal
MAP3K7 Tumor
MAP3K7 Normal
PSTPIP2 Tumor
PSTPIP2 Normal
PYCARD Tumor
PYCARD Normal
RIPK1 Tumor
RIPK1 Normal

D TGCT

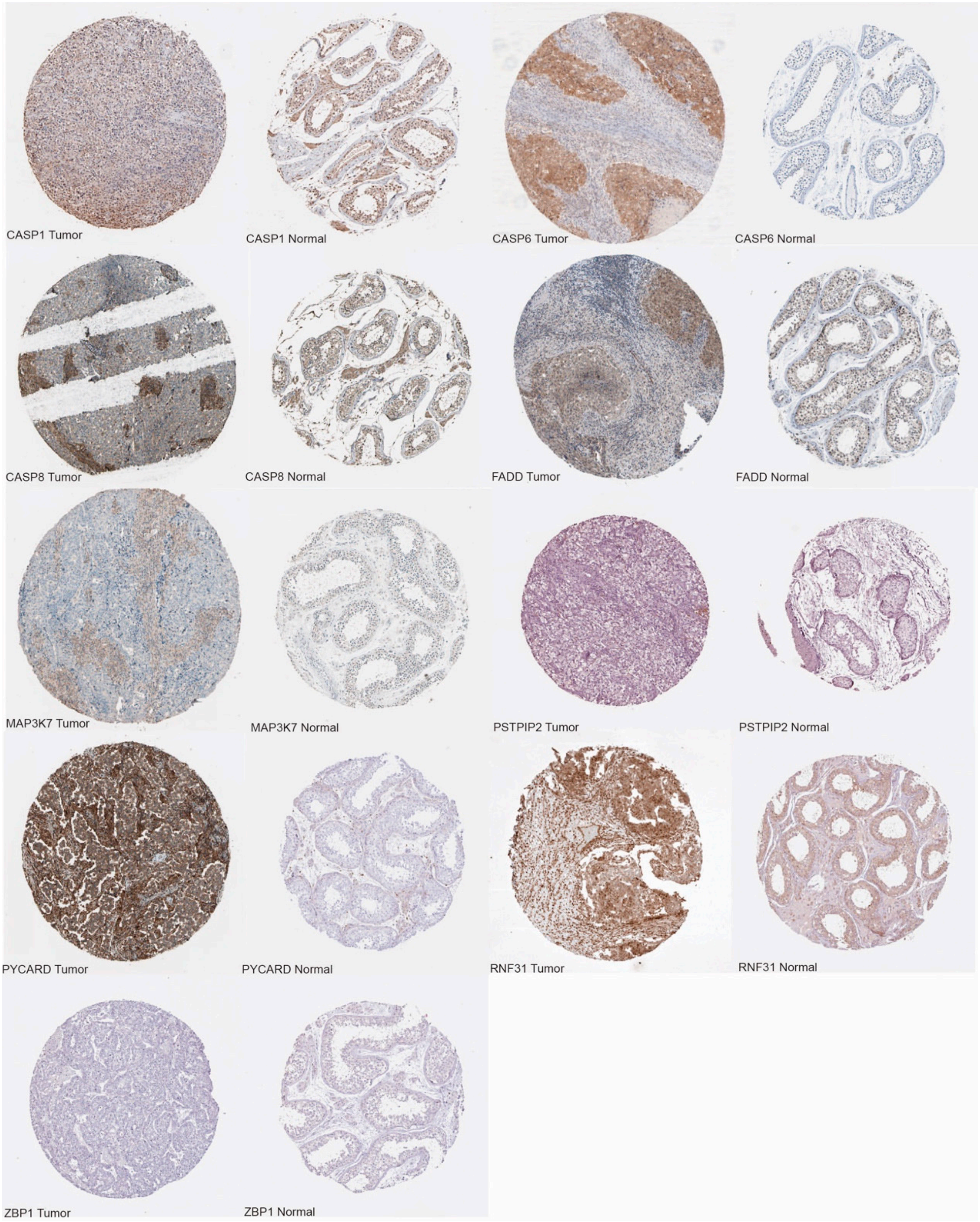

CASP1 Tumor
CASP1 Normal
CASP6 Tumor
CASP6 Normal
CASP8 Tumor
CASP8 Normal
FADD Tumor
FADD Normal
MAP3K7 Tumor
MAP3K7 Normal
PSTPIP2 Tumor
PSTPIP2 Normal
PYCARD Tumor
PYCARD Normal
RNF31 Tumor
RNF31 Normal
ZBP1 Tumor
ZBP1 Normal

E

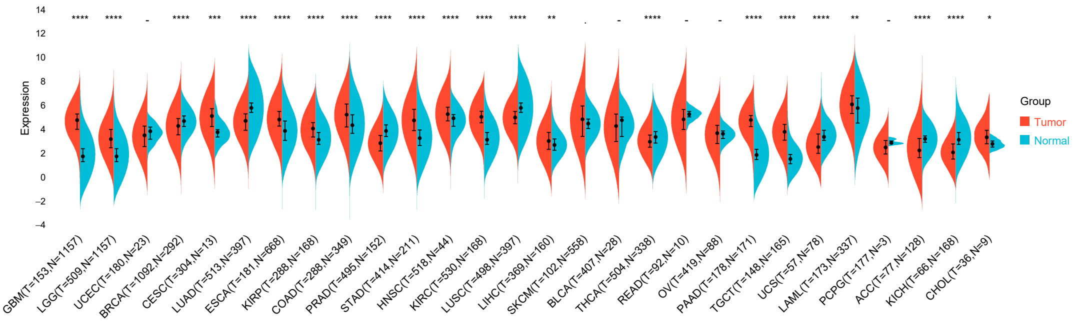

Expression
14
12
10
8
6
4
2
0
−2
−4
**** **** - **** *** **** **** **** **** **** **** **** **** **** ** . - **** - - **** **** **** ** - **** **** *
GBM(T=153,N=1157)
LGG(T=509,N=1157)
UCEC(T=180,N=23)
BRCA(T=1092,N=292)
CESC(T=304,N=13)
LUAD(T=513,N=397)
ESCA(T=181,N=668)
KIRP(T=288,N=168)
COAD(T=288,N=349)
PRAD(T=495,N=152)
STAD(T=414,N=211)
HNSC(T=518,N=44)
KIRC(T=530,N=168)
LUSC(T=498,N=397)
LIHC(T=369,N=160)
SKCM(T=102,N=558)
BLCA(T=407,N=28)
THCA(T=504,N=338)
READ(T=92,N=10)
OV(T=419,N=88)
PAAD(T=178,N=171)
TGCT(T=148,N=165)
UCS(T=57,N=78)
LAML(T=173,N=337)
PCPG(T=177,N=3)
ACC(T=77,N=128)
KICH(T=66,N=168)
CHOL(T=36,N=9)
Group
Tumor
Normal

Supplementary Figure S1. (A)-(D) Histochemical expression of PANRGs in glioma, KIRC, PAAD, STAD, and TGCT. (E) The expression level of CSP1 in 28 cancers.

Supplementary Figure S2

Age

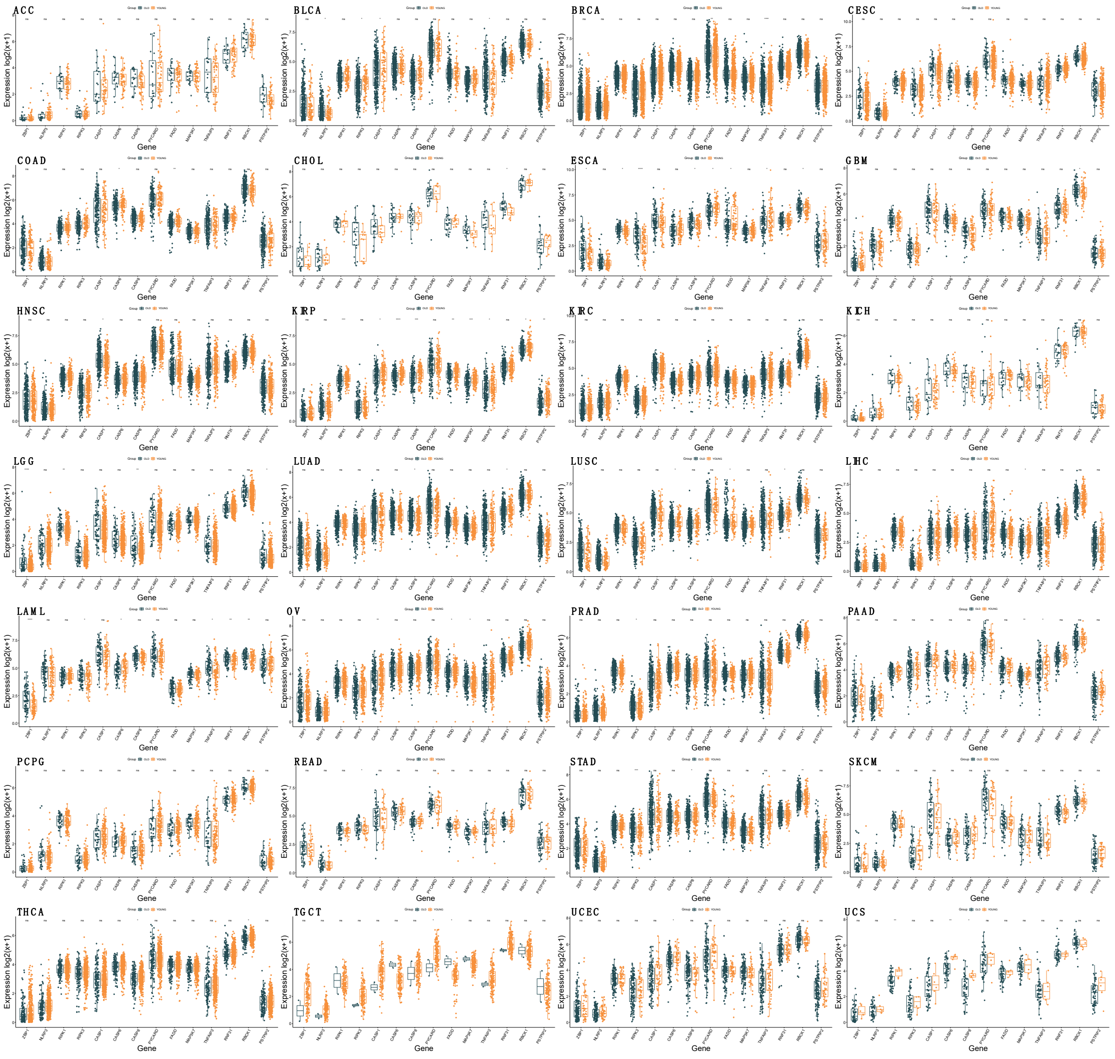

Stage

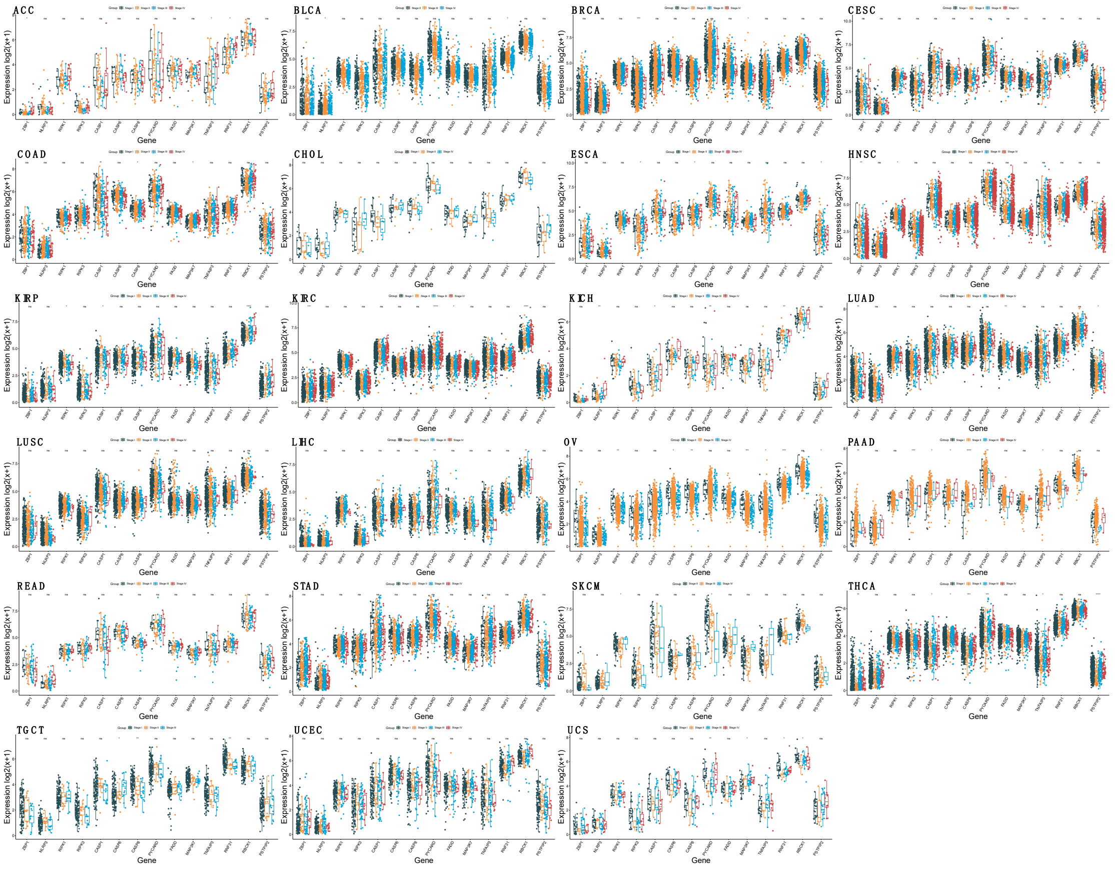

N stage

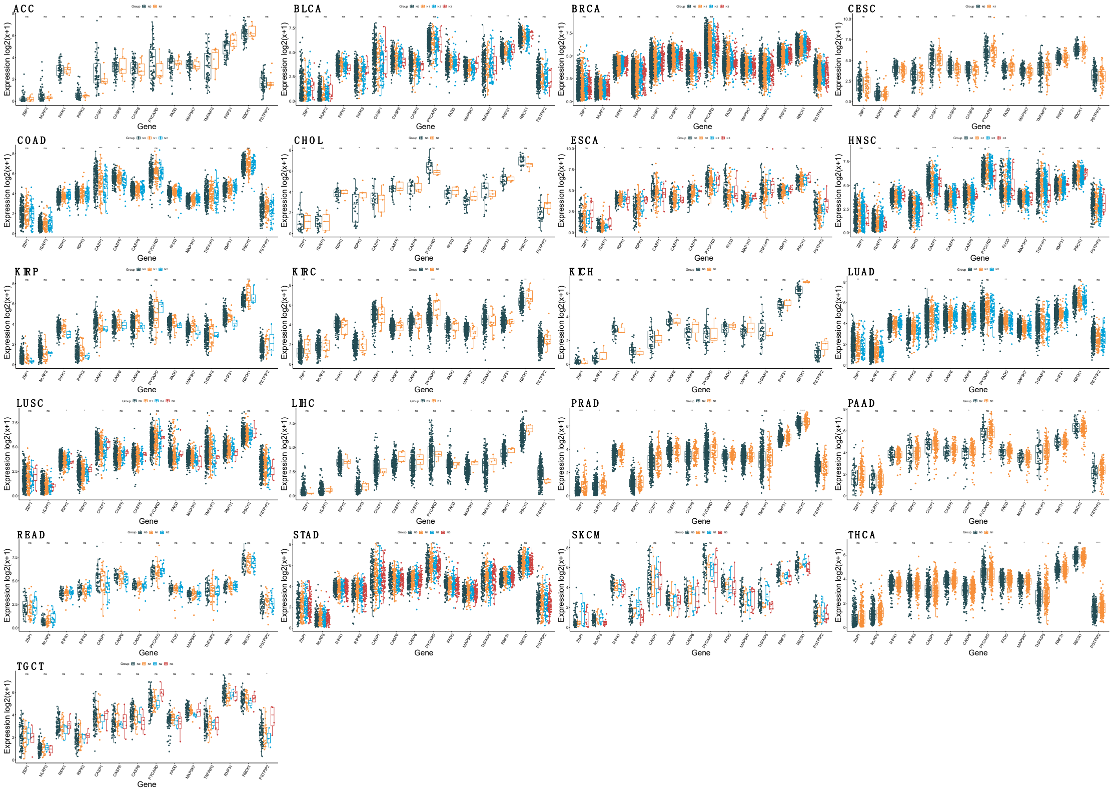

M stage

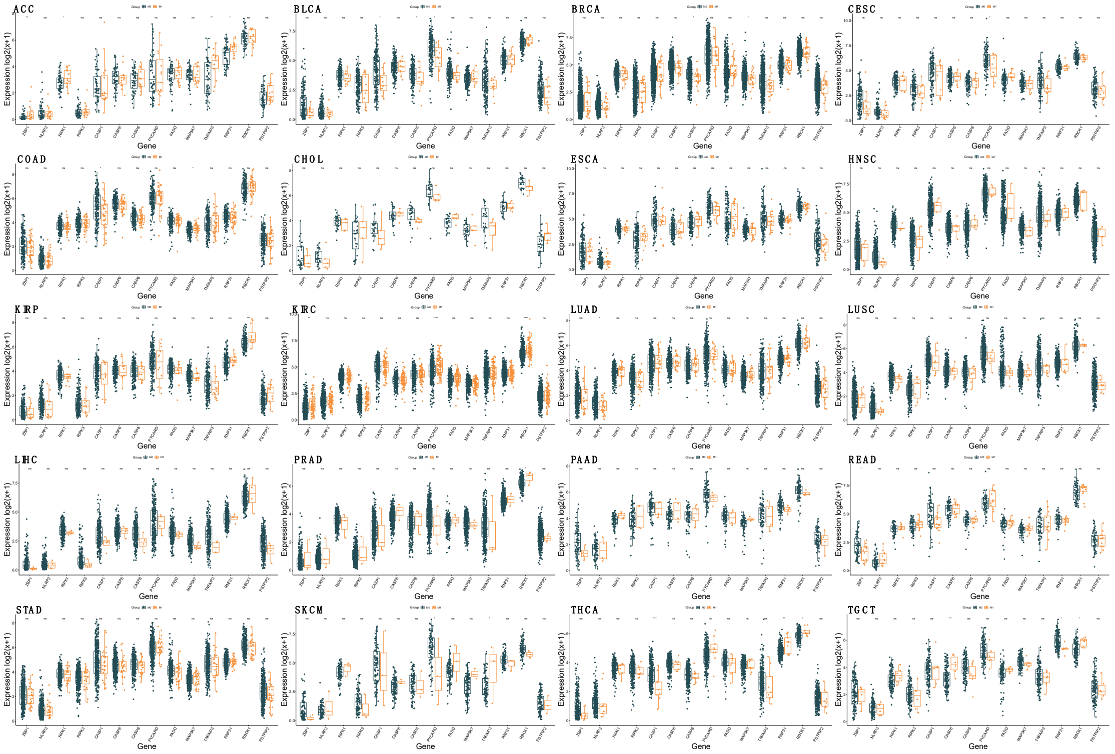

T stage

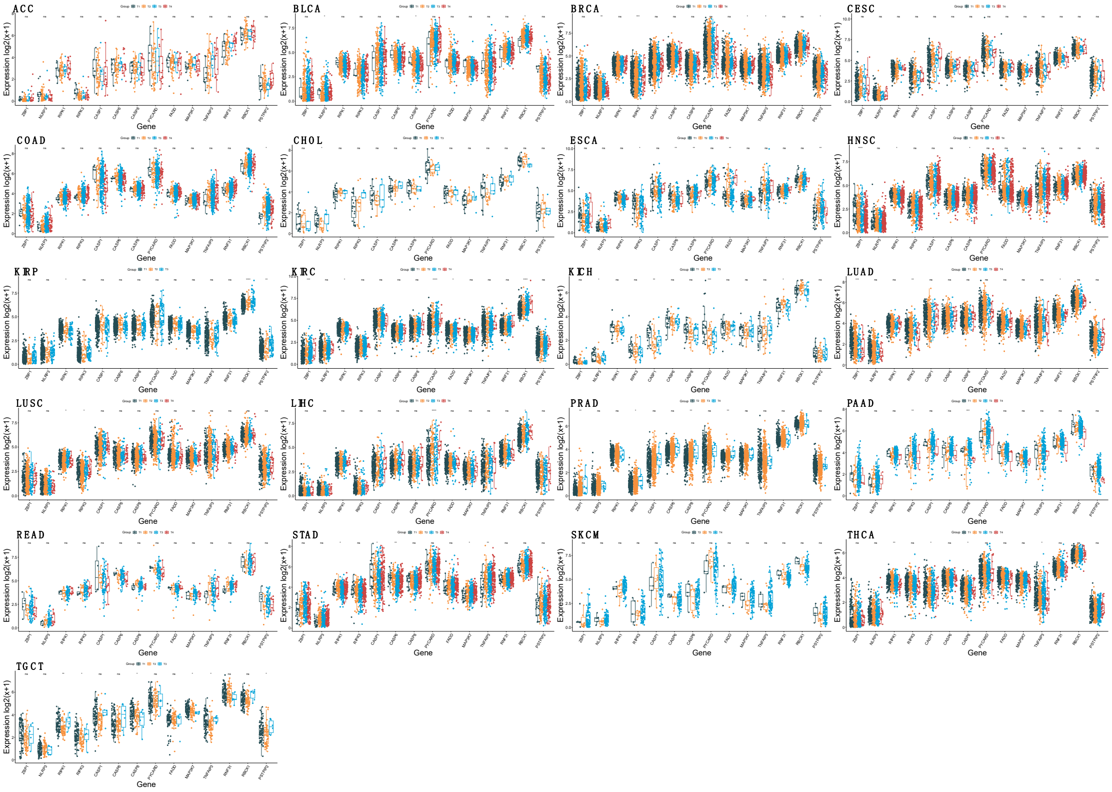

Grade

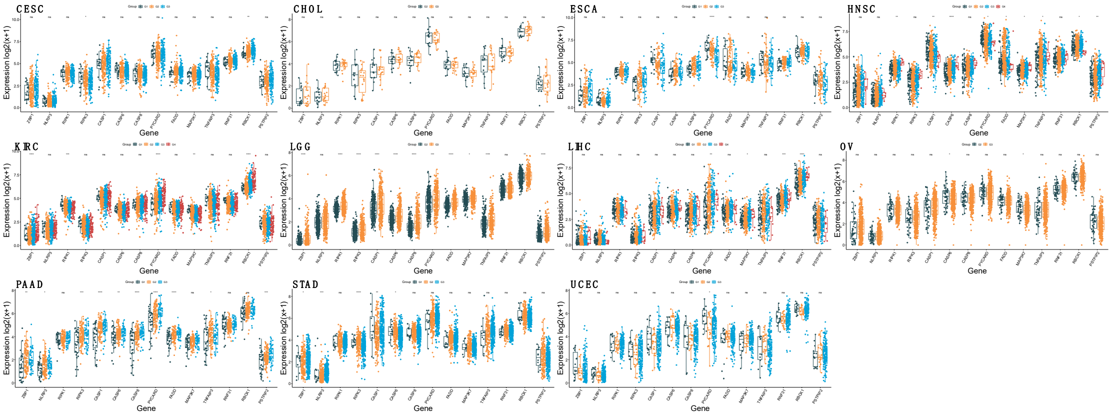

Gender

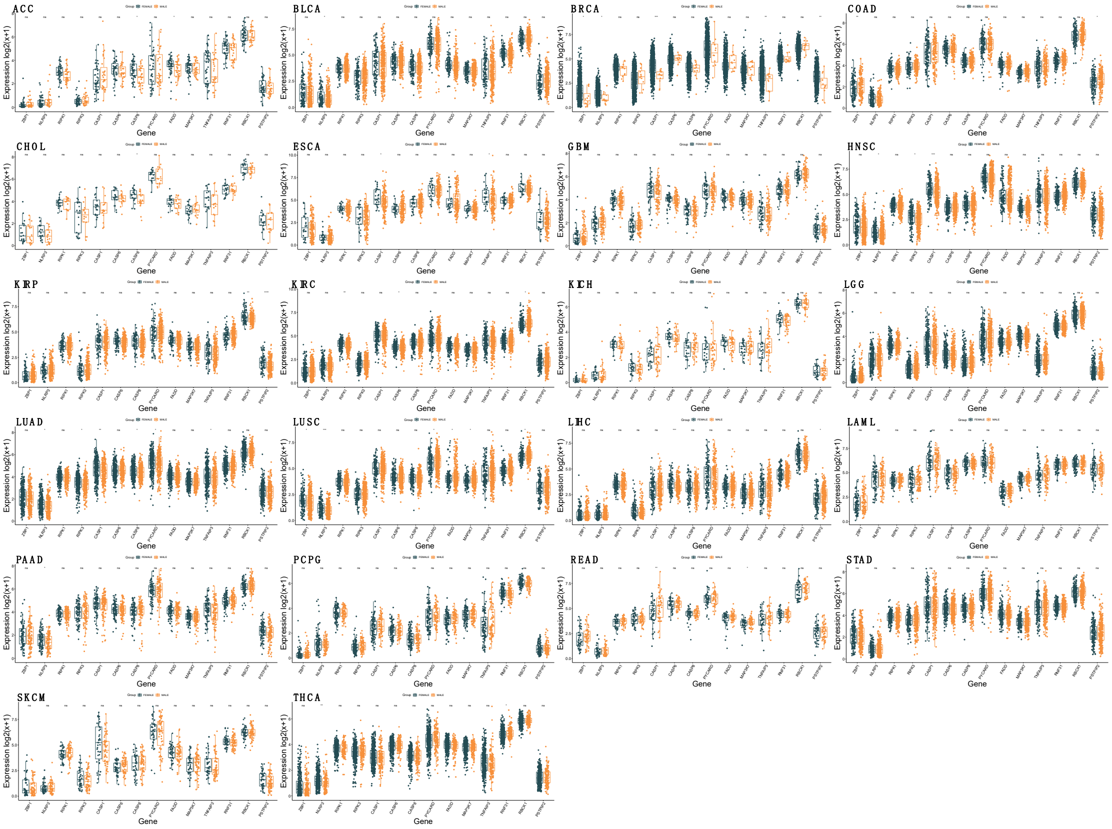

Supplementary Figure S3

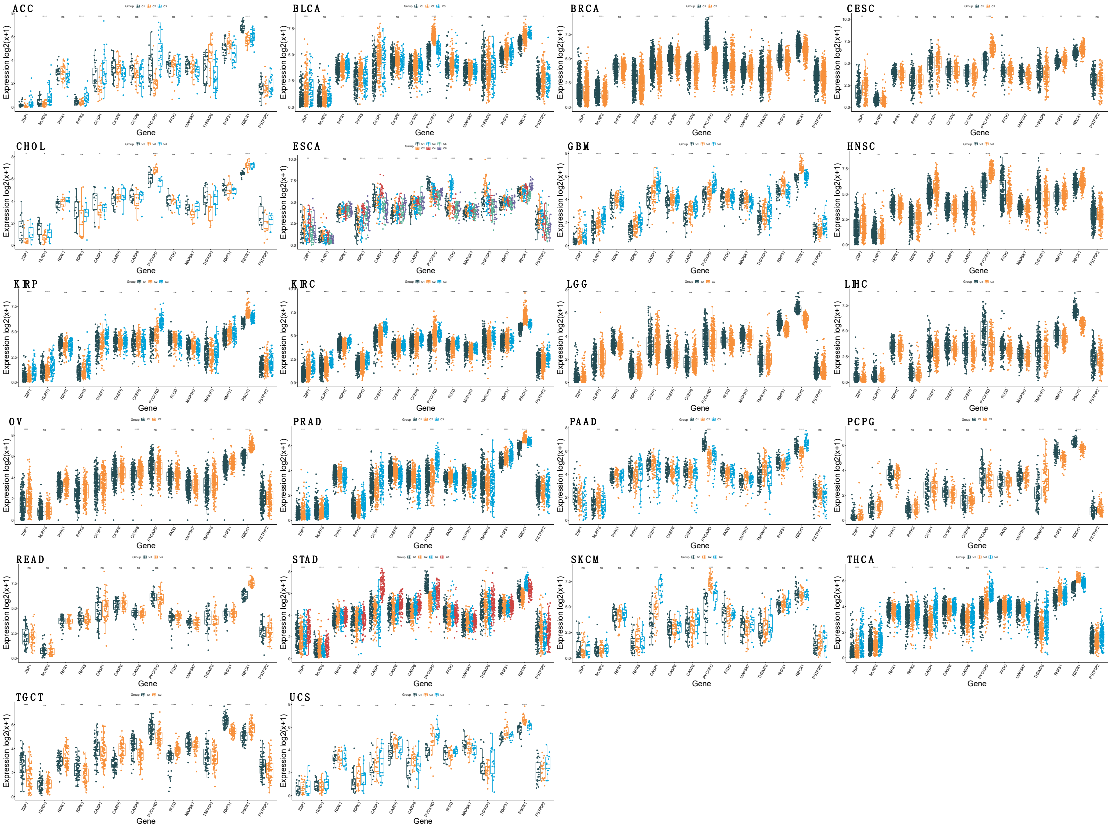

Supplementary Figure S3. Cluster analysis of PANRGs in various tumors.

Supplementary Figure S4

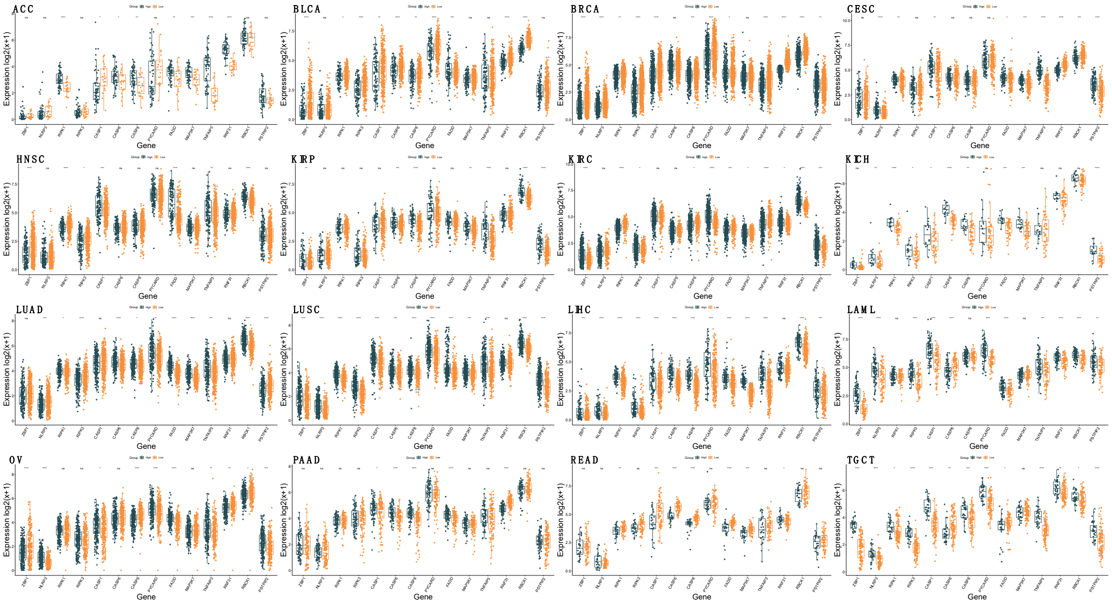

Supplementary Figure S4. The differential expression of genes in various tumors according to their high and low groupings.

Supplementary Figure S5

A

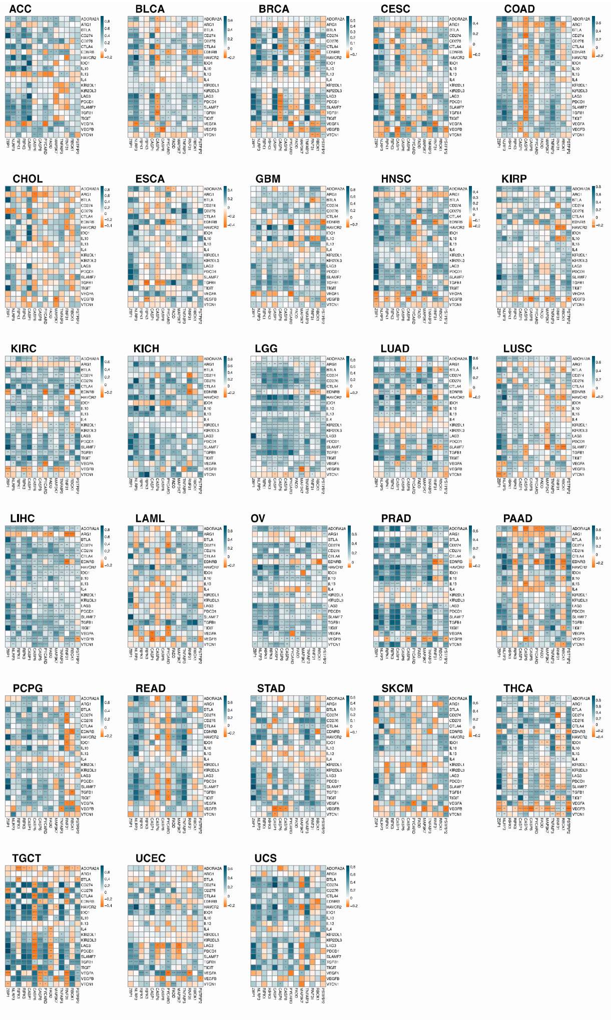
ACC
BLCA
BRCA
CESC
COAD
CHOL
ESCA
GBM
HNSC
KIRP
KIRC
KICH
LGG
LUAD
LUSC
LIHC
LAML
OV
PRAD
PAAD
PCPG
READ
STAD
SKCM
THCA
TGCT
UCEC
UCS

B

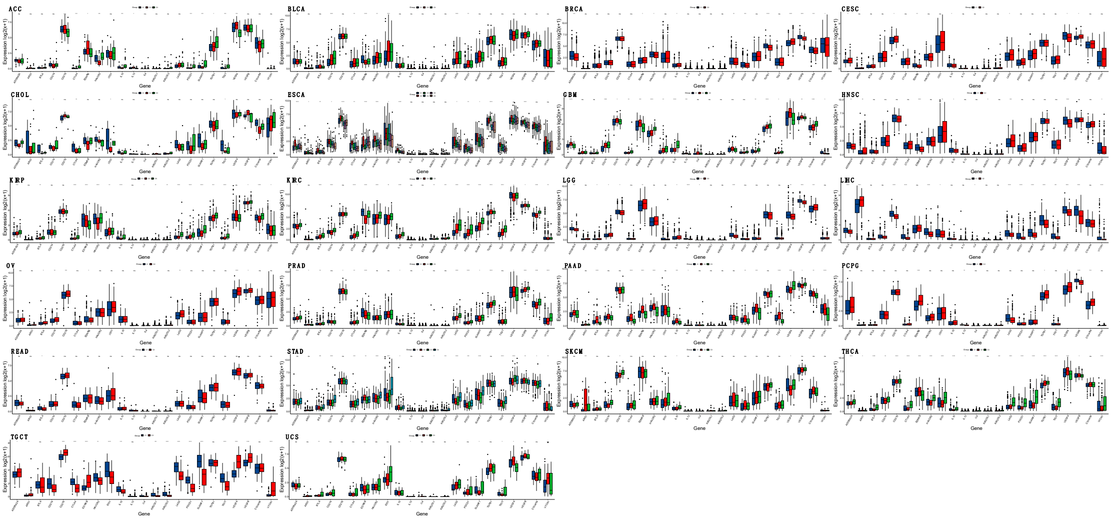
ACC
BLCA
BRCA
CESC
CHOL
ESCA
GBM
HNSC
KIRP
KIRC
LGG
LIHC
OV
PRAD
PAAD
PCPG
READ
STAD
SKCM
THCA
TGCT
UCS
Gene

C

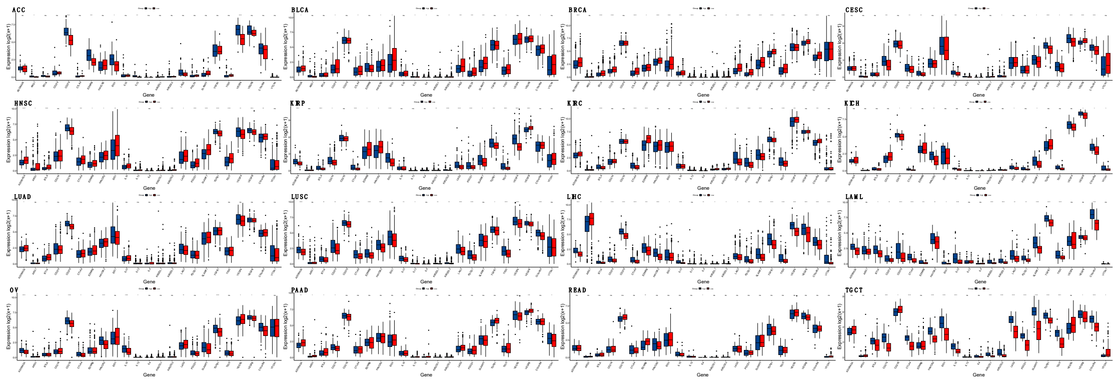

Supplementary Figure S5. (A)The linear relationship between gene set and immune checkpoint inhibitory. (B) Differences in clustering of immune checkpoint inhibitors in different tumors. (C) Differences in lasso of immune checkpoint inhibitors in different tumors.

Supplementary Figure S6

A

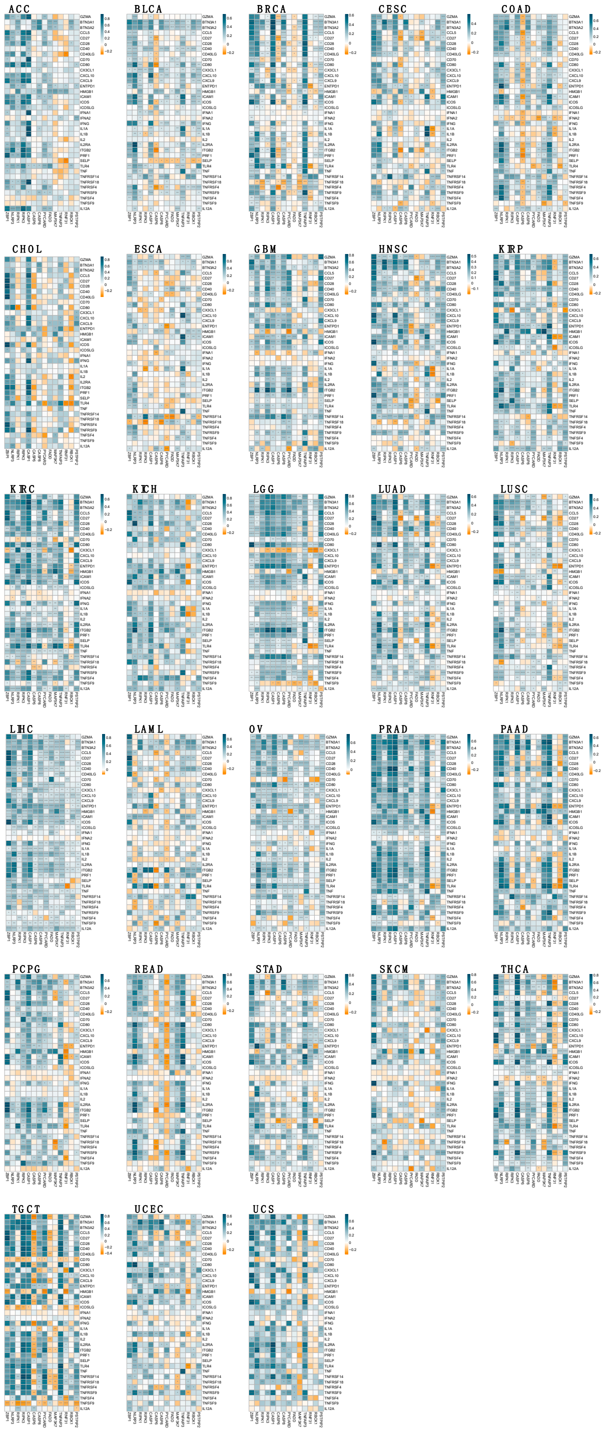

B

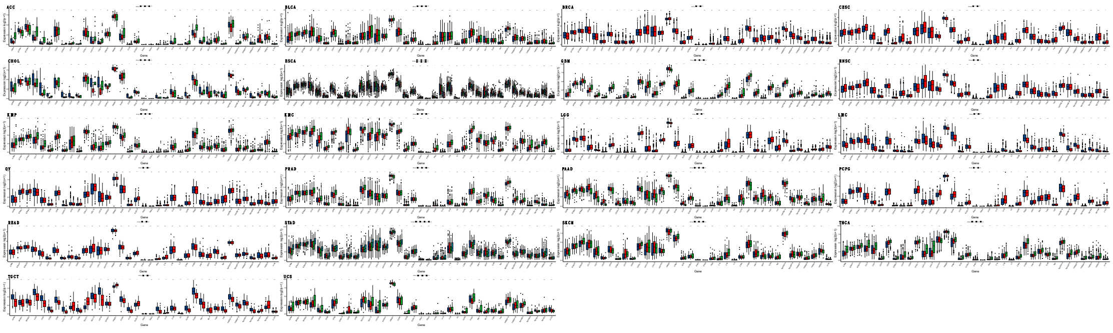

C

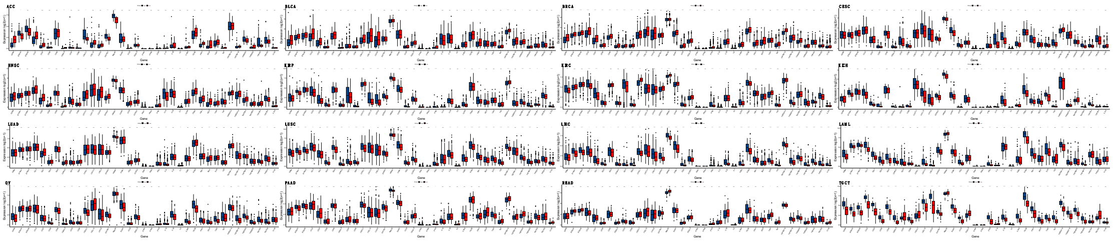

Supplementary Figure S6. (A)The linear relationship between gene set and immune checkpoint stimulatory. (B) Differences in clustering of immune checkpoint stimulators in different tumors. (C) Differences in lasso of immune checkpoint stimulators in different tumors.

Supplementary Figure S7

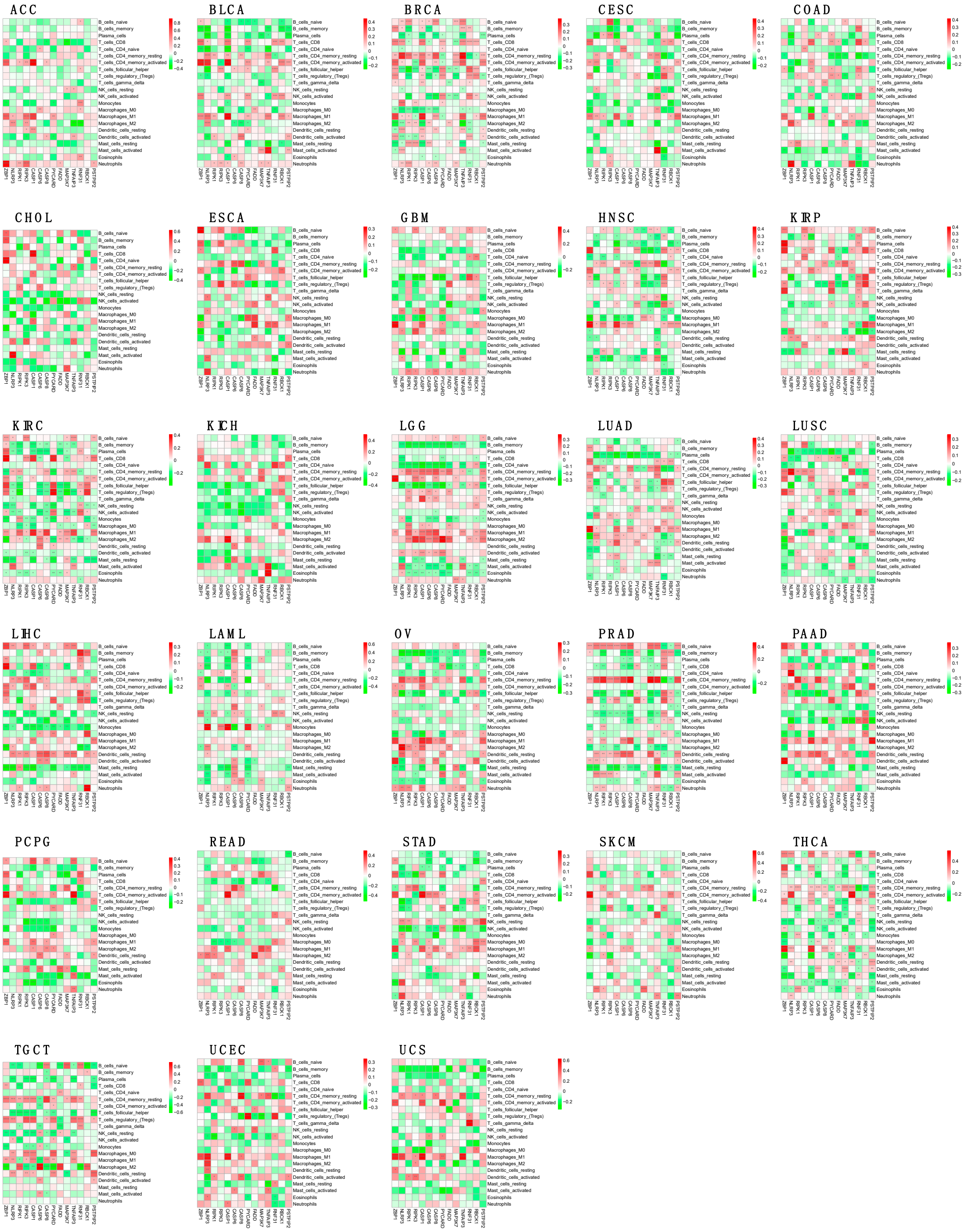

Supplementary Figure S7. The relationship between PANRGs and immune infiltrating cells in pan cancer.

Supplementary Figure S8

A

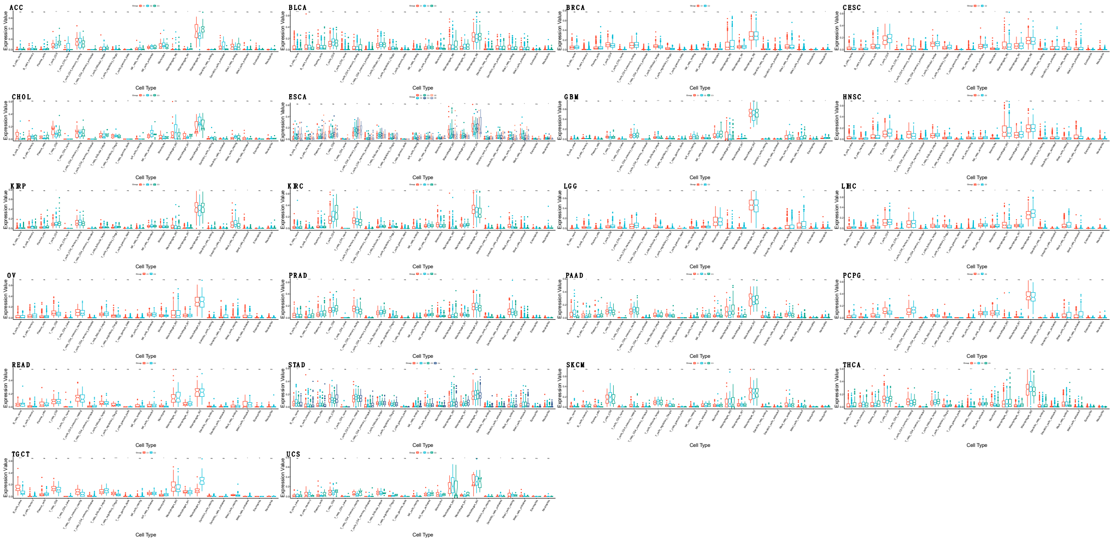

B

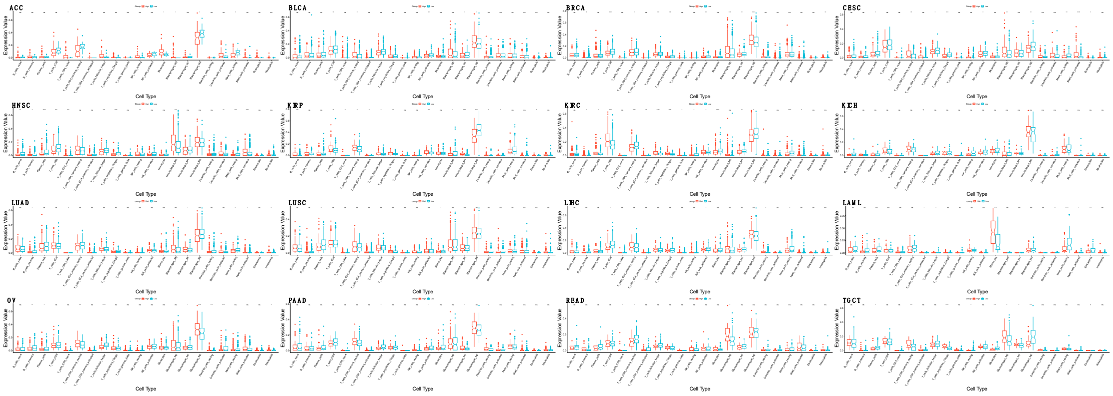

Supplementary Figure S8. (A) Differences in clustering between PANRGs and immune cells in pan cancer. (B) Differences in lasso between PANRGs and immune cells in pan cancer.

Supplementary Figure S9

A

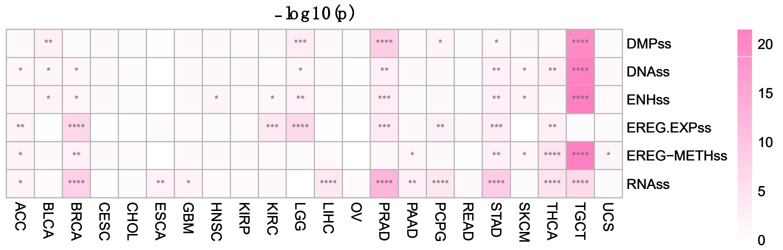

B

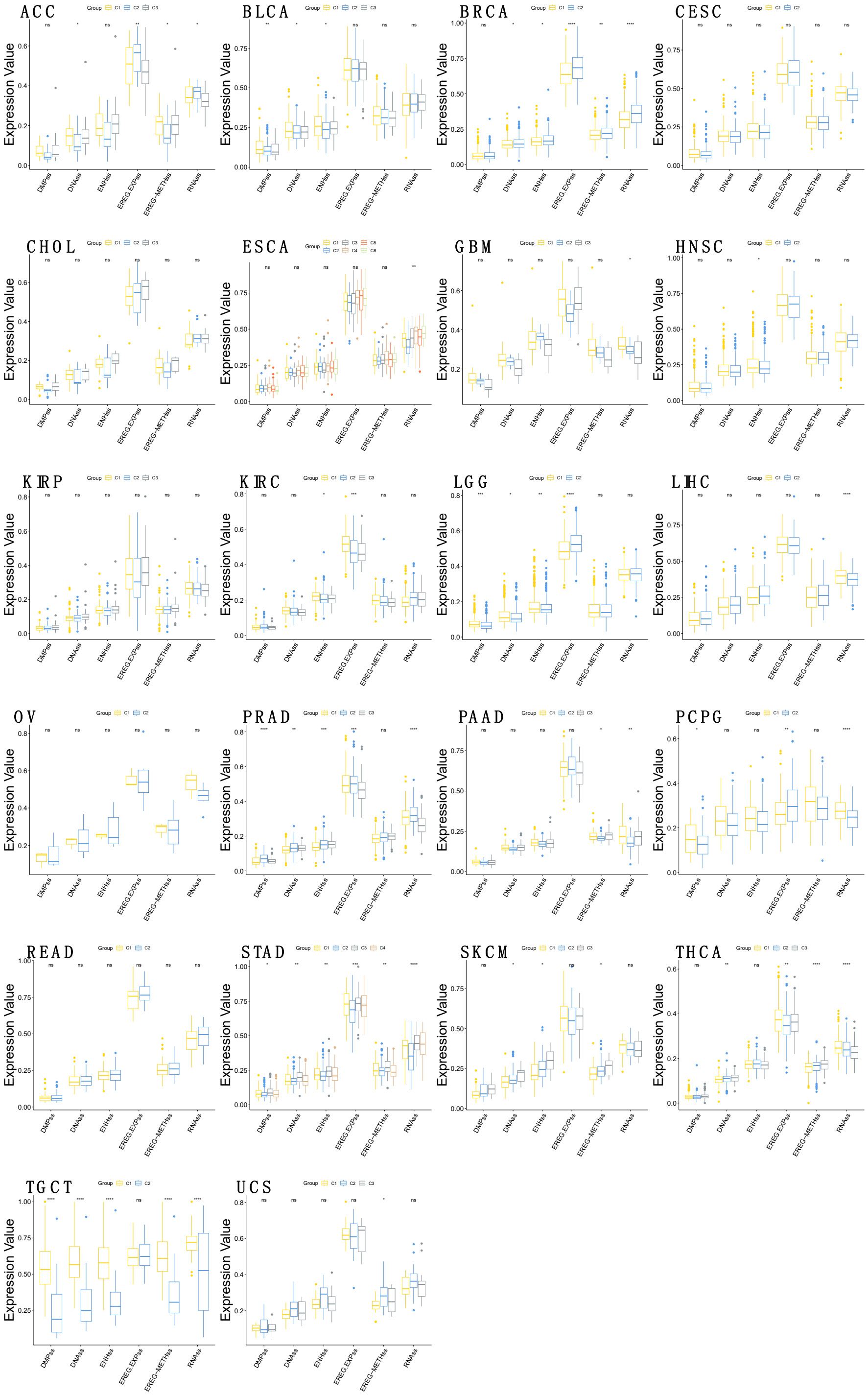
ACC
BLCA
BRCA
CESC
CHOL
ESCA
GBM
HNSC
KIRP
KIRC
LGG
LIHC
OV
PRAD
PAAD
PCPG
READ
STAD
SKCM
THCA
TGCT
UCS
Expression Value
Group
DMPss
DNAss
ENHss
EREG.EXPss
EREG-METHss
RNAss

C

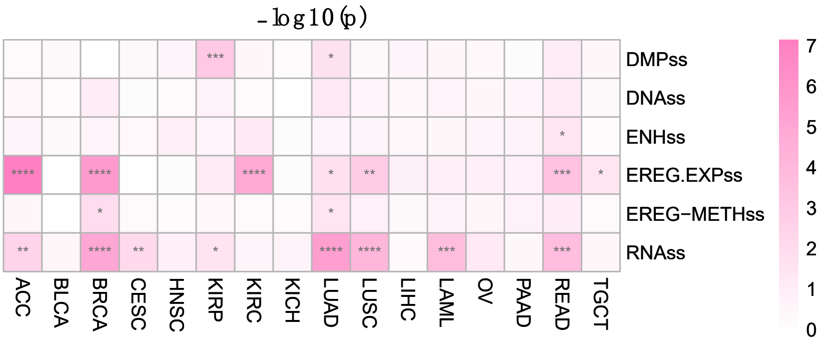

-log10(p)
DMPss
DNAss
ENHss
EREG.EXPss
EREG-METHss
RNAss
ACC
BLCA
BRCA
CESC
HNSC
KIRP
KIRC
KICH
LUAD
LUSC
LIHC
LAML
OV
PAAD
READ
TGCT
0
1
2
3
4
5
6
7

D

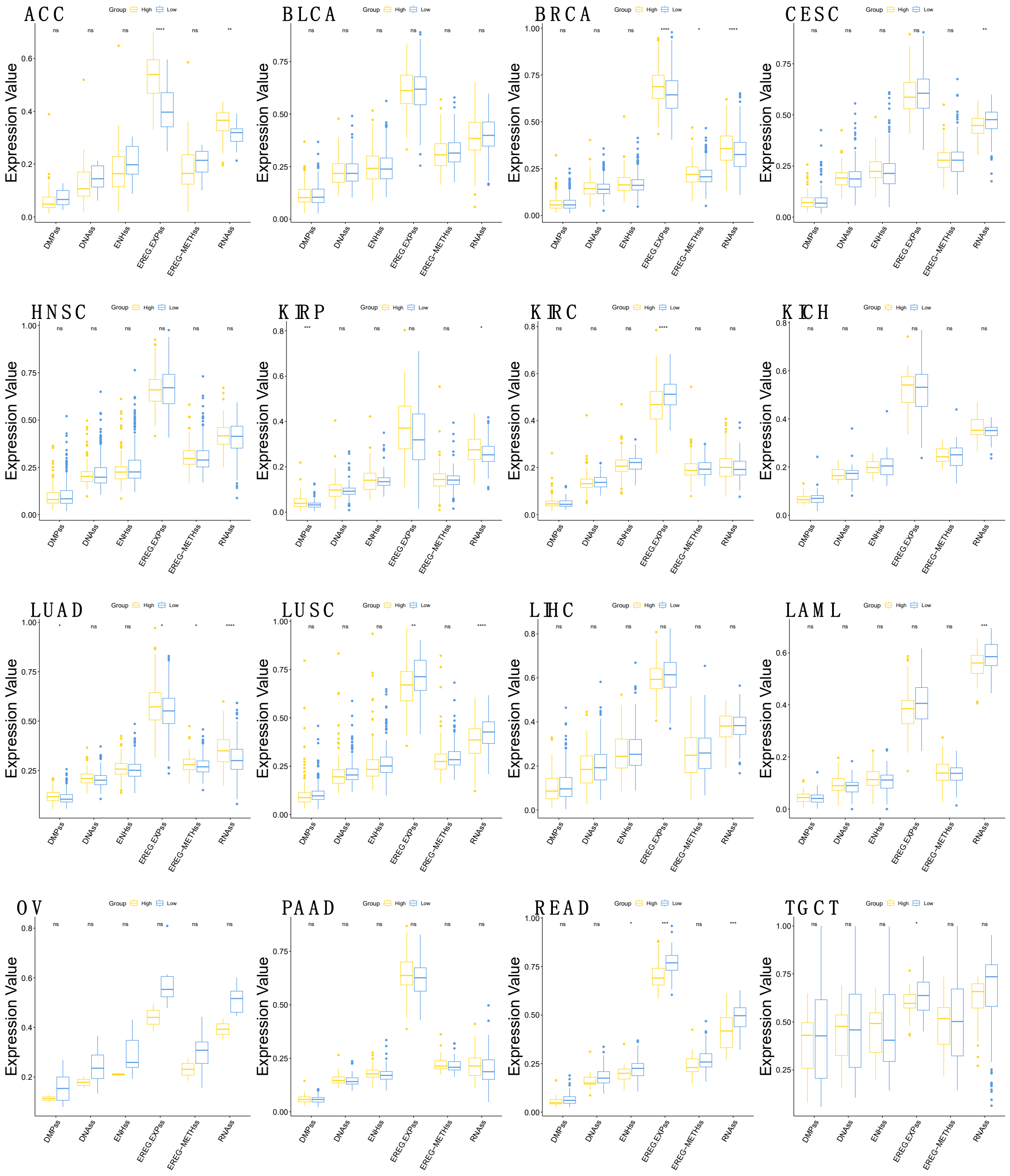

ACC
BLCA
BRCA
CESC
HNSC
KIRP
KIRC
KICH
LUAD
LUSC
LIHC
LAML
OV
PAAD
READ
TGCT
Group High Low
Expression Value
DMPss
DNAss
ENHss
EREG.EXPss
EREG-METHss
RNAss

Supplementary Figure S9. (A) and (B) Differences in clustering of tumor stemness. (C) and (D) Differences in lasso of tumor stemness.

Supplementary Figure S10

A

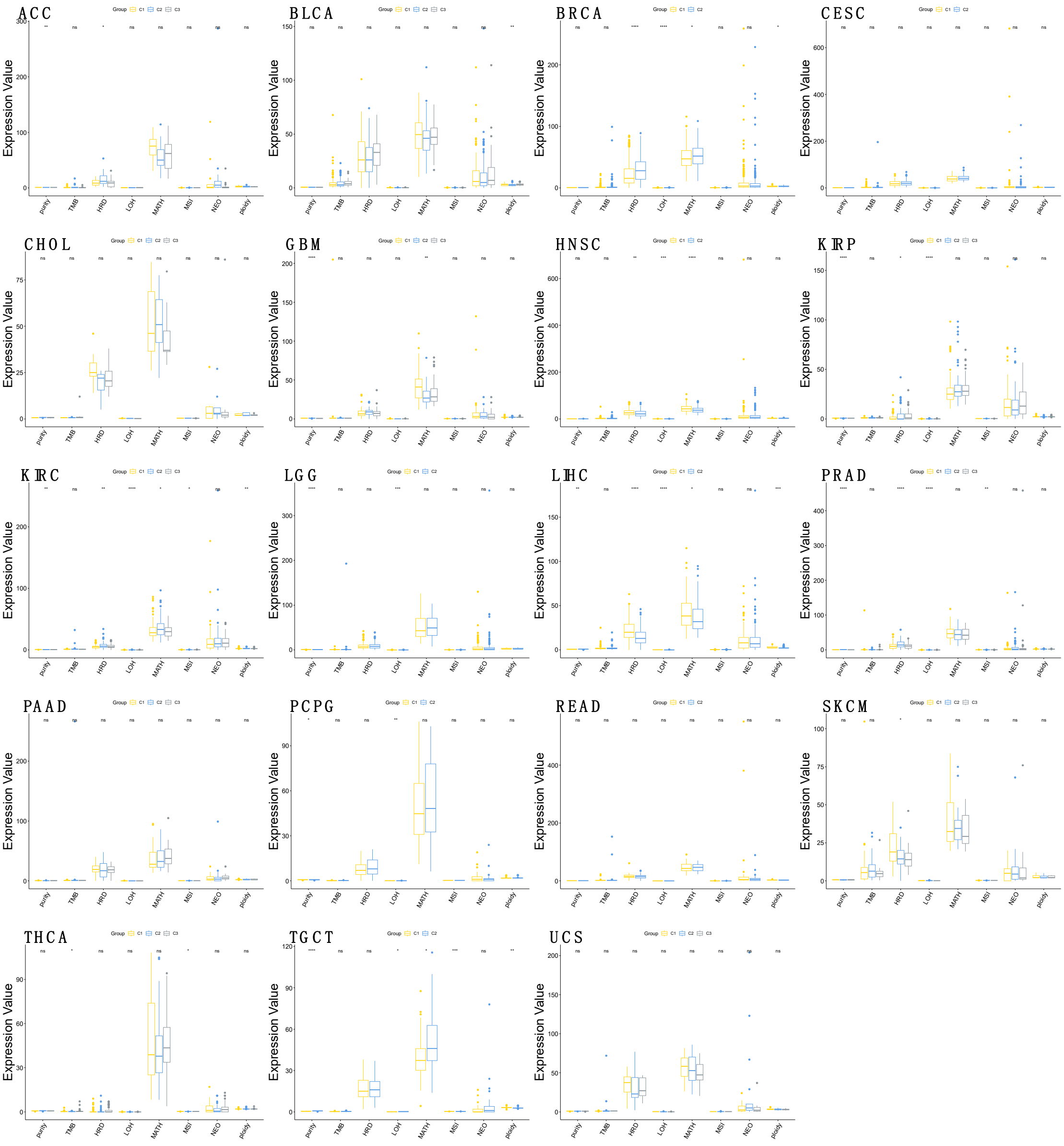

B

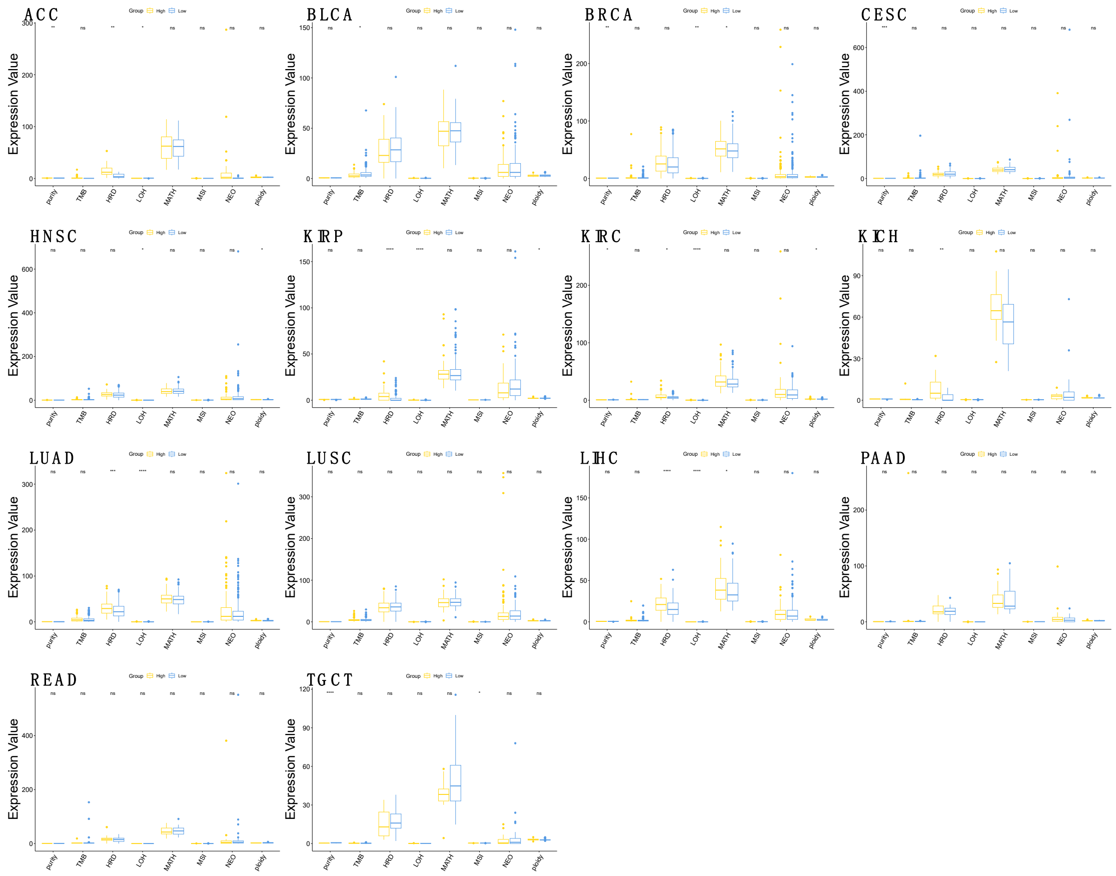

Supplementary Figure S10. (A) Differences in clustering of tumor heterogeneity among PANRGs in pan cancer. (B) Differences in lasso of tumor heterogeneity among PANRGs in pan cancer.
